# Vacuum assistance therapy as compared to early reconstructive treatment in deep sternal wound infection

**DOI:** 10.1177/1457496920979289

**Published:** 2020-12-16

**Authors:** E. Hämäläinen, J. Laurikka, H. Huhtala, O. Järvinen

**Affiliations:** 1 Faculty of Medicine and Health Technology, Tampere University, Tampere, Finland; 2 Department of Cardiothoracic Surgery, Tampere Heart Hospital, Tampere, Finland; 3 Faculty of Social Sciences, Tampere University, Tampere, Finland

**Keywords:** Cardiac surgery, deep sternal wound infection, DSWI, mediastinitis, pressure therapy, candida

## Abstract

**Background and Aims:**

Deep sternal wound infection is a major concern after cardiac surgery. This study describes the incidence of postoperative deep sternal wound infections after cardiac surgery and compares two available treatment modalities.

**Materials and Methods:**

In Tampere University Hospital, 7973 open heart operations were performed between 2007 and 2016. Patients treated for a postoperative deep sternal wound infection were categorized in two groups based on treatment: revision surgery with early reconstruction (revision group; 74 patients) or vacuum-assisted closure treatment (VAC group; 55 patients). The end points in comparisons were overall mortality and hospitalization time.

**Results:**

A total of 129 patients (1.6%) developed a postoperative deep sternal wound infection. The 30-day mortality rates were 8.1% and 3.6%, the 90-day mortality rates were 15.5% and 18.2%, and the 1-year mortality rates were 17.6% and 23.6% for the revision and VAC group, respectively. There was no statistically significant difference in mortality rates. The hospital stay was 18 days in the revision group and 38 days in the VAC group (p < 0.001). The secondary intensive care unit stay was longer in the VAC group (median 1 vs 4, p = 0.011). The most common pathogens isolated in the first reoperation were coagulase-negative staphylococci (33.8% and 41.8%, respectively; p = 0.366), and positive candida findings were more common in the VAC group (4.1% vs 37.0 %, p < 0.001).

**Conclusion:**

Vacuum-assisted closure treatment induces an inferior outcome in terms of fungal infections, treatment times, and the number of reoperations.

## Introduction

Postoperative deep sternal wound infection (DSWI) is a severe complication and is often associated with other infections. These include sternitis and pulmonary infections, which makes the condition even more severe (1, 2). The occurrence of a DSWI has been reported in 0.4% and 2.5% of the cases (3, 4). There are several known independent risk factors for DSWI, such as age, diabetes mellitus (DM), obesity (high body mass index (BMI)), renal failure, the use of both internal thoracic arteries as bypass grafts, and a reoperation for bleeding and chronic obstructive pulmonary disease (COPD) (5). DSWI is associated with increased 1-year mortality, which can be as high as 25.4% (6). Higher age, a high EuroSCORE, combination surgery, left ventricular insufficiency, multiple organ failure, and the duration of the surgery all increase the mortality associated with DSWI (6). However, the rate of DSWIs has been decreasing along with the development of surgical techniques (7).

The aim of this study was to evaluate two available surgical treatment modalities for DSWIs in a single cardiac surgical institution from 2007 to 2016: early revision with reconstruction and vacuum-assisted closure (VAC). The outcome measures were mortality, hospitalization time, and length of treatment in the intensive care unit (ICU), as well as the number of reoperations and the microbiological wound and blood culture findings during wound care. Revision treatment included the revision and closure or the revision and continuous irrigation of the closed mediastinum. If VAC was used as the primary treatment for DSWI, it was followed by a secondary closure of the wound. Furthermore, microbiological findings were evaluated.

## Materials and Methods

The data were obtained from all 7673 patients who underwent open heart surgery at Tampere University Hospital between 1 January 2007 and 31 December 2016. All patient data were collected from the patient records of the Heart Center at Tampere University Hospital, which were the primary data source for our study. Further identification was carried out in connection with the hospital’s infection register to identify all cases with a DSWI. Finally, the data included 129 DSWI patients. Microbiological sample findings obtained during the first reoperation or blood culture findings between the primary operation and the first reoperation were collected. Pathogens were categorized into six groups: *Staphylococcus aureus*, coagulase-negative staphylococci (CoNS), polymicrobial, *Enterococcus faecalis, Streptococcus*, and other pathogens. Patients with an initially negative microbiological finding at the first reoperation or blood culture were regarded negative even if samples taken later showed pathogenic bacteria. To avoid bias, we did not include later bacterial findings because of possible contamination. The criteria for DSWI were clinical status and a positive blood/wound culture or a macroscopic infection in the mediastinum. Candida findings were also collected. In the revision group, candida was considered positive if the finding was obtained after the surgery, and in the VAC group, it was considered positive if the sample was positive after pressure therapy was administered.

The preoperative protocol for elective patients included an outpatient clinic visit during which the patient’s teeth were checked, any beard was planned to be trimmed, and blood glucose levels were controlled. Subsequently, dental problems were treated before cardiac surgery, and the patient’s body and hair were washed with a chlorhexidine-containing detergent. The protocol for antibiotic prophylaxis in cardiac surgery entailed intravenous single-dose cefuroxime (3 g) as an induction antibiotic 1 h before the incision, followed by supplementary doses administered every 4 h for as long as the surgery continued. In patients allergic to cefuroxime, 1 g (2 g if weight above 100 kg) of vancomycin was given, and supplementary doses were provided every 12 h during the surgery. In patients with methicillin-resistant *Staphylococcus aureus* (MRSA), the induction antibiotic was a combination of cefuroxime and vancomycin. Cardiac surgery was performed through a midline sternotomy and completed with a sternal saw and a division of the pericardium. The continuation of antibiotic therapy after surgery for prolonged prophylaxis of 2 days was decided by the operating surgeon on a case-by-case basis, depending on the patient’s infection risk. The most common risk factors that supported the continuation of antibiotic treatment were extreme obesity, unbalanced diabetes, immunosuppressive medications, the use of both internal mammary arteries, skin infections, complicated surgery, or a considerably lengthy duration of surgery.

After the surgery, all patients were treated overnight in the ICU before being transferred to the hospital ward when they had achieved a stable hemodynamic, respiratory, and neurological status.

The primary choice of antimicrobial treatment in a DSWI was intravenous cefuroxime 1.5 g administered three times daily. It was started empirically on all patients whose clinical picture was susceptible to DSWI. Later, antimicrobial treatment was allocated to the specific microbe according to the blood culture response. The antimicrobial treatment was continued intravenously for 4 weeks. If the clinical picture and laboratory tests showed no sign of infection after 4 weeks, the antimicrobial treatment was not continued orally.

There were two general treatment modalities in use for DSWIs at Tampere Heart hospital during the 10-year study period. Starting from the beginning of 2007, surgeons mostly used a treatment that included re-sternotomy, intravenous antibiotics, and sternum rewiring, sometimes accompanied with substernal saline flushing with drains. Later during the study period, VAC therapy became more common and was eventually adopted as a general treatment for DSWI. It included re-sternotomy, the installation of the VAC sponges, and replacing them every 2 days. Both therapy options were used simultaneously at the surgeon’s preference.

For analysis, the patients were divided into two groups according to the treatment modality. If the patient was treated with revision surgery, he or she was categorized into the revision group (74 patients). If the treatment was accomplished using the VAC technique, the patient was categorized into the VAC group (55 patients). Patient characteristics were expressed as a percentage of the total in both groups.

Surgical operations were divided into three groups. The coronary artery bypass grafting (CABG) group included all patients who underwent isolated CABG (n = 81). Patients who underwent a single-valve procedure were categorized into the Valve group (n = 18). All other patients were placed in the Other/Combination category (n = 30).

All statistical analyses were performed using IMB SPSS statistics for Windows, Version 23.0 (IBM Corp., Armonk, NY, USA). Categorical variables were analyzed with the chi-square test and Fisher’s exact test. Continuous variables were analyzed using the Mann–Whitney U-test. Kaplan–Meier analyses with the log-rank test were used for comparisons of mortality between groups.

## Results

There were 103 male (79.8%) and 26 female patients (20.2%) in the study. The age range was from 40 to 87 years, and the median age was 71 years. The median ages in the two treatment groups were similar (71.5 years in the revision group and 71 years in the VAC group). Patient characteristics in the two groups are listed in Table 1, and there were no statistically significant differences between the treatment groups.

**Table 1:** Patient characteristics in the treatment groups.

	Revision		VAC		*p*-value
	n = 74		n = 55		
	n	%	n	%	
Male	59	79.7	44	80.0	1.000
Procedure					0.428
CABG	50	67.6	31	56.4	
Valve	9	12.2	9	16.4	
Other/combination	15	20.3	15	27.3	
Obesity (BMI ⩾30 kg/m^2^)	28	38.4	25	45.5	0.471
Urgency					0.613
Elective	33	44.6	27	49.1	
Urgent/emergency	41	55.4	28	50.9	
Smoker	17	23.0	14	28.0	0.807

VAC: vacuum-assisted closure; CABG: coronary artery bypass grafting; BMI: body mass index.

The annual occurrence of DSWIs was 1.6% throughout the 10-year study period. There was no systematic increase or decrease in annual rates, and they showed only random variation between 1.22% and 2.42%.

Among the operations performed between 2007 and 2016, a CABG procedure was performed on 52.3%, a valve operation on 19.3%, and other/combination surgery on 28.4% of the patients. Of the DSWIs, 62.8% appeared after CABG operations, 14.0% after valve operations, and 23.3% after other/combination procedures. Sixty operations (46.5%) resulting in a DSWI were performed electively, 38 (29.5%) were urgent, and 31 (24.0%) were emergency procedures. The time interval between the initial operation and the diagnosis of a DSWI showed no statistical difference between the revision group and the VAC group (median 12 vs 11 days, respectively; p = 0.506).

The most common pathogens to cause DSWIs were CoNS (37.2%). *S. aureus* was the second most common pathogen, isolated in 32.6% of the cases. There was no statistical difference between the treatment groups in the isolated microbiological findings. In both groups, the most common pathogen isolated in the first reoperation was CoNS, which was found in 33.8% and 41.8% of the cases in the revision and VAC group, respectively. *S. aureus* was the second largest pathogen group isolated and was found in 32.4% and 32.7% of the cases, respectively. Candida sample findings were significantly less often positive in the revision group than in the VAC group (4.1% vs 37.0%, p < 0.001) (Table 2).

**Table 2:** Principal microbe isolations in the treatment groups.

	Revision		VAC		*p*-value
	n = 74		n = 55		
	n	%	n	%	
Bacterial findings					0.324
CoNS	25	33.8	23	41.8	
S. aureus	24	32.4	18	32.7	
Polymicrobial	4	5.4	3	5.5	
*Enterococcus faecalis*	1	1.4	2	3.6	
Streptococcus	2	2.7	1	1.8	
Other	1	1.4	3	5.5	
Negative	17	23.0	5	9.1	
Candida	3	4.1	20	37.0	< 0.001

VAC: vacuum-assisted closure; CoNS: coagulase-negative staphylococci

**Fig 1.**
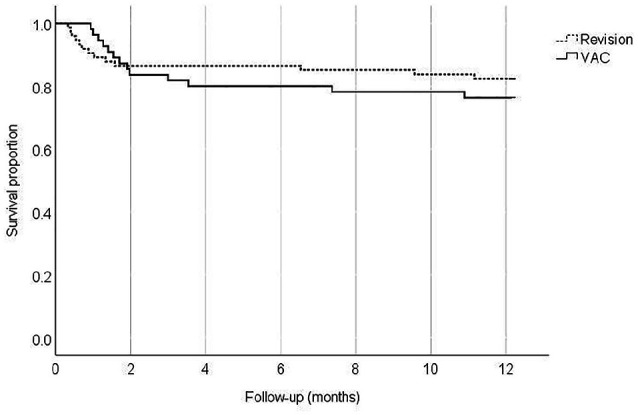
Postoperative 12-month survival of patients in the treatment groups (p = 0.470).

During the 10-year study period, the 30-day mortality associated with DSWIs was 6.2%, whereas the 90-day mortality was 15.5% and the 1-year mortality was 20.2%. When compared between the study groups, the 30-day mortality showed no difference, with six (8.1%) deaths occurring in the revision group and two (3.6%) in the VAC group. The 90-day mortality was 13.5% in the revision group and 18.2% in the VAC group, and the 1-year mortality was 17.6% in the revision group and 23.6% in the VAC group. There was no overall difference in mortality between these groups, log-rank p = 0.470 (Fig. 1). The urgency of the surgery was related to survival. In the DSWI group, mean survival after the operation was better if the operation was performed electively than if the surgery was urgent or emergent (7.7 years vs 6.1 years, p = 0.053).

Patients treated by means of revision had a shorter stay on the university hospital ward (median 18 vs 38 days in the VAC group, p < 0.001). The primary stay in the ICU was longer when the patient was treated with VAC (median 1 vs 2 days, p = 0.045), and if readmitted to the ICU, the patients who were treated with VAC had a longer stay than those included in the revision group (median 1 vs 4 days, p = 0.011). Furthermore, the number of reoperations was higher in the VAC group than in the revision group (median 1 vs 7, p < 0.001) (Table 3).

**Table 3:** Length of hospital stay and stay in secondary intensive care, expressed as days, in the treatment groups.

	Revision		VAC		*p*-value
	n = 74		n = 55		
	Median	Q_1_-Q_3_	Median	Q_1_-Q_3_	
University hospital	18	13-23	38	27-51	< 0.001
Secondary ICU	1	1-4	4	1-9.5	0.011

VAC: vacuum-assisted closure; ICU: intensive care unit.

The use of plastic surgical flaps for sternal reconstruction was analyzed between the groups. Flaps were more commonly used in the VAC group (59.6% vs 40.4%, p = 0.001). There was also a statistically significant difference in the type of tissue used as a flap. The most common flap used in the revision group was an omentum flap (78.9%) and in the VAC group a pectoralis major flap (84.2%) (p < 0.001).

## Discussion

The aim of the study was to evaluate the annual occurrence of DSWIs, to detect potential reasons for variations in the occurrence, and to compare the two different treatment modalities for postoperative DSWIs (revision surgery and VAC). The end points used as primary parameters in the comparison between these groups were mortality, length of stay in the university hospital, and ICU stay.

Although the incidence of postoperative DSWIs is low, it is a serious condition and may lead to death. In our material, the annual rates varied from 1.22% to 2.4% and showed no systematic change between 2007 and 2016. This finding is in conflict with a previous study which showed an earlier systematic decrease in the incidence of mediastinitis in 2005 (7). This could be explained by the differences in surgical techniques after the study period of Finkelstein et al. and attributed to increased efforts in the prevention of hospital-acquired infections over the decades.

In the study by Eklund et al. (8), a DSWI extended the postoperative hospital stay by an average of 13 days. Consequently, it causes more physiological and psychological stress (9) as well as extra costs, which are reported to be as high as US$62,773, in addition to the normal cost of the surgery (3).

The age of patients selected for open heart surgery is increasing, leading to an increased risk of death after the operation (6). Also, during the last few decades, an increasing number of heart procedures have been performed using minimally invasive cardiological techniques, which has led to a reduction in open heart surgery, mainly in coronary artery procedures, and may have led to a more challenging patient population undergoing open heart surgery. However, our study population was fairly homogeneous between 2007 and 2016, which may also explain the steady rate of DSWIs (Table 1). Although more coronary artery revascularizations are performed with endovascular techniques, CABG was more common in patients with a DSWI than in the whole population undergoing open heart surgery between 2007 and 2016 in our institution. This could be explained by the fact that the use of internal thoracic artery as a bypass graft is common in CABG procedures and it may slightly increase the risk of infection, as it diminishes the perfusion in sternal tissue (5).

Different treatment techniques have been examined to find the safest possible care in the case of postoperative DSWIs. Still, the treatment protocol varies between institutions (10). A postoperative DSWI has classically been treated with intravenous antibiotics, re-sternotomy, continuous irrigation of the mediastinum, and, if necessary, reconstruction with omentum or muscle flaps (11). Recently, many institutions, including our own, have adopted negative pressure wound therapy (VAC) as primary care for a postoperative DSWI. It has been reported to improve breathing, to reduce the stay in ICU, and to have a decreasing effect on mortality among patients suffering from a postoperative DSWI (5, 12). In contrast, the study by Risnes et al. shows no difference in long-term survival between the VAC group and the classical treatment group after post-CABG mediastinitis (13).

In our patient population, we could not detect any significant difference in mortality between the treatment groups at any observation point. It seems that the 1-year survival is lower in the VAC group, but the result is not statistically significant. The result may be clinically relevant, however, and the lack of statistical significance could be due to the limited size of the study population. Still, our results do not show a better outcome in patients who were treated with VAC when compared with patients in the revision group. Thirty-day survival is better with VAC, but 90-day survival and 1-year survival are poorer when a patient is treated with VAC. In the short term, VAC seems to be a better treatment option, but in the long term, it somehow turns out to be unfavorable as regards patient survival. This could be explained by the fact that, during VAC treatment, the patient is exposed to the physical and psychological stress of multiple reoperations, which could lead to a faster decline in the quality of life and the ability to function. Moreover, the VAC sponge generally needs to be replaced every 2 days, which compromises patient nutrition, as the patient cannot eat until the VAC sponge has been changed in the operation theater. Subsequently, a compromised nutritional status slows the patients’ rehabilitation from a DSWI.

One factor which may explain negative pressure therapy not being better is the significantly higher incidence of fungal infections. During VAC treatment, the chest is left open, which causes an increased risk of fungal infections, with other related complications in the long term. This leads to slower healing and, therefore, longer hospitalization due to the delay in the secondary reconstruction of the sternal wound. Due to the high incidence of candida infections during VAC treatment, the surgeons should consider whether antifungal medications, such as fluconazole, should be routinely started earlier for patients with prolonged VAC treatment and subsequent reoperations. Future research should also focus on these contaminations, since there appears to be a correlation between long VAC treatment and fungal infections during the open treatment of a sternal wound infection.

The length of stay both in the university hospital and the ICU was longer and the number of reoperations higher if the patient was treated with VAC. The higher number of reoperations in the VAC population compared to the revision group is explained by the fact that the VAC sponge needs to be changed every 2–3 days to remove the debris. However, the prolonged stay in the ICU and the higher number of reoperations cause more physical and psychological stress for the patients, which may lead to psychological disorders and, consequently, slower healing and rehabilitation after mediastinitis (14). Also, the higher number of reoperations and the prolonged ICU stay increase the cost of the treatment. Considering the higher cost of the treatment and the lack of a significant favorable effect on mortality and hospitalization time, it is necessary to question VAC as a primary treatment for mediastinitis. It should be considered whether VAC is, indeed, the best choice of treatment in all cases of DSWI or whether there are identifiable risk factors that are in favor of treatment with primary revision and closure instead of VAC.

The common pathogens to cause mediastinitis are part of the normal skin flora, mostly *Staphylococcus* species. The most common pathogen has been found to be *Staphylococcus epidermidis*, which has been isolated in more than 50% of all mediastinitis cases (11). Our findings are in line with the previous study, and CoNS were the most common pathogens in microbiological samples obtained during the first reoperation in both treatment groups. *S. aureus* was the second most common pathogen in both groups. There were more negative findings during the primary reoperation in the revision group, which could be caused by the earlier inclusion of antibiotics in the treatment protocol or even by less advanced microbiological techniques or processes at the beginning of the study period.

The strength of our study is the long and complete follow-up in terms of survival and the relatively large study population compared to some other studies comparing these surgical methods. An important clinical observation was the large difference in candida culture findings between the revision and VAC group. The limitations of the study include the fact that the two treatment modalities were not used during the same chronological periods and that the preoperative preparations of the surgical procedure have also changed over the years. VAC was adopted as the first-line treatment later in the study period, and consequently, in the later phase of our observation period, the DSWI patients were more likely to have been treated with VAC therapy. However, the overall patient characteristics were similar in both groups (Table 1).

Our study reveals a fairly steady rate of DSWIs after cardiac surgery in single-center tertiary-level hospital care in Northern Europe. When we compared revision and closure to VAC treatment, we could not detect a significant change in mortality, but we did observe a significantly longer treatment time and higher rate of fungal colonization in connection with VAC treatment. These observations should be noted in the care of postoperative cardiac patients.
